# Advancements in smart healthcare in emergency trauma care: from intelligent triage to prognostic prediction

**DOI:** 10.3389/fpubh.2026.1834849

**Published:** 2026-06-02

**Authors:** Fanyi Cheng, Tao Xu, Junwei Mao, Zhiping Li, Yajun Wang

**Affiliations:** Department of Emergency Medicine, People's Hospital of Quzhou, Quzhou, Zhejiang, China

**Keywords:** artificial intelligence, diagnostic imaging, emergency trauma care, machine learning, prehospital triage, prognostic prediction, smart healthcare, telemedicine

## Abstract

Emergency trauma care demands rapid, accurate decisions across the continuum from prehospital triage to definitive treatment and follow-up. Smart healthcare technologies, particularly artificial intelligence (AI) and telemedicine, are increasingly being integrated into trauma systems to augment clinical judgment, improve resource allocation, and expand access to specialist expertise. This mini-review synthesizes recent evidence on the application of these technologies in emergency trauma care. Current studies suggest that AI can support prehospital triage, identify life-threatening injuries on imaging, predict mortality, transfusion needs, and other adverse outcomes, and facilitate early risk stratification for both physiological and psychological sequelae. Telemedicine has shown value in remote consultation, transfer decision support, and virtual follow-up, with potential to reduce unnecessary interfacility transfers and optimize trauma system efficiency. However, the field remains constrained by major challenges, including the predominance of retrospective validation studies, limited prospective evidence of clinical utility, concerns regarding algorithmic opacity and bias, and persistent barriers related to workflow integration, infrastructure, reimbursement, and human factors. Overall, smart healthcare is reshaping emergency trauma care from intelligent triage to prognostic prediction, but its translation into routine practice will require prospective implementation studies, explainable and equitable model development, and closer alignment with real-world clinical workflows.

## 1 Introduction

Emergency trauma care represents one of the most time-sensitive and resource-intensive domains within modern medicine. The “golden hour” principle underscores the critical importance of rapid, accurate decision-making from the point of injury through definitive care to optimize survival and functional outcomes. However, this continuum is fraught with challenges, including geographical disparities in access to specialist expertise, cognitive overload on clinicians, and the inherent difficulty of predicting dynamic patient trajectories based on limited early data (1, 2). In response to these pressures, the integration of smart healthcare technologies, primarily artificial intelligence (AI) and telemedicine, has emerged as a transformative force with the potential to revolutionize trauma systems (3, 4). This evolution aims not merely to automate tasks but to augment human clinical judgment, enhance system efficiency, and ultimately improve patient-centric outcomes.

Artificial intelligence, encompassing machine learning (ML) and deep learning (DL), offers unprecedented capabilities in pattern recognition from complex, high-dimensional data (5). In trauma care, this translates to applications ranging from the analysis of prehospital physiological waveforms to the automated detection of life-threatening injuries on medical imaging and the prediction of complications or mortality (6, 7). Concurrently, telemedicine has matured from a niche communication tool to a strategic platform for delivering specialist knowledge across geographical barriers (4, 8). It enables remote evaluation, triage decision support, and guidance for interventions at non-specialist centers, thereby potentially reducing unnecessary patient transfers and optimizing the use of centralized trauma resources (9, 10). The convergence of these technologies promises a more responsive, efficient, and equitable trauma care ecosystem.

This mini-review synthesizes the latest evidence on the application of smart healthcare across the trauma care continuum. It critically examines the progression from intelligent prehospital triage and automated diagnostics to advanced prognostic modeling for both physiological and psychological outcomes. Furthermore, it delves into the current gaps in evidence, methodological controversies surrounding AI transparency and bias, and the practical barriers to clinical implementation. By evaluating both the demonstrated potential and the persisting challenges, this review aims to provide a balanced perspective on the current state of the field and outline informed directions for future research and integration (Table 1). Unlike prior reviews that have primarily summarized isolated applications of AI or telemedicine, this mini-review is organized along the trauma care continuum and emphasizes the translational interface between technical capability and clinical utility. In particular, we focus on how smart healthcare tools may influence destination triage, imaging workflow, escalation of care, transfer decisions, and longitudinal follow-up, while also examining the barriers that currently limit their real-world implementation.

**Table 1:** Representative applications, clinical value, and current challenges of smart healthcare technologies in emergency trauma care.

Technology and stage	Representative applications	Potential clinical value	Current challenges
AI Prehospital triage	ML-based field triage models predict severe trauma, shock, transfusion need, and requirement for definitive surgical care (11–14, 44).	May improve destination decisions, earlier trauma-team activation, and prearrival risk stratification at the point of first contact.	Most models remain retrospective; performance may decline across settings because of limited external validation and workflow integration.
AI Imaging support	CNN-based tools assist in the detection of intracranial hemorrhage, rib and limb fractures, and abdominal organ injuries on radiography or CT (16–26).	Can prioritize urgent imaging review, reduce missed injuries, and support earlier escalation of time-sensitive management.	False positives/negatives, interoperability with PACS, technical downtime, and limited prospective impact data remain concerns.
AI Outcome prediction	Models estimate mortality, frailty-related outcomes, and massive transfusion requirements; some are embedded in point-of-care tools (27–34).	May support escalation decisions, hemorrhage-pathway activation, ICU triage, and more targeted allocation of limited resources.	Calibration, transparency, and fairness are variable; many tools lack evidence that they improve real-world decisions or outcomes.
AI Long-term sequelae	Emerging models screen for post-traumatic stress disorder risk and other neurobehavioral consequences after acute trauma (35, 36).	May enable earlier identification of patients needing psychological surveillance, referral, and structured post-discharge follow-up.	Evidence is still early-stage, with uncertain generalizability and limited implementation data in routine trauma systems.
Telemedicine Transfer triage	Remote review supports triage and helps identify patients who do not require interfacility transfer, including pediatric and hand trauma cases (10, 37, 38).	Can support transfer/no-transfer decisions, reduce avoidable interfacility movement, and preserve regional trauma capacity.	Effectiveness depends on local staffing, connectivity, reimbursement, and acceptance by referring clinicians.
Telemedicine Remote consultation	Virtual specialist input can guide diagnosis, imaging review, and acute management at spoke hospitals or rural facilities (8, 9, 39).	Extends specialist input to underserved settings and may improve diagnostic and management decisions when local expertise is limited.	Robust evidence for hard outcomes such as mortality or diagnostic accuracy remains limited and context-dependent.
Telemedicine Virtual follow-up	Virtual fracture clinics and post-discharge follow-up pathways extend trauma care beyond the emergency department (40–43).	May support risk-appropriate follow-up, reduce unnecessary in-person visits, and improve outpatient resource allocation.	Patient selection, digital literacy, safeguarding, and data security must be addressed for sustainable implementation.

AI, artificial intelligence; CNN, convolutional neural network; CT, computed tomography; ML, machine learning; PACS, picture archiving and communication system.

### 1.1 Literature identification and selection

This mini-review was based on a targeted search of PubMed and Web of Science for studies published between January 2020 and February 2026. Search terms combined concepts related to trauma and emergency care with terms related to smart healthcare technologies, including artificial intelligence, machine learning, deep learning, telemedicine, remote consultation, triage, imaging, and prognostic prediction. We prioritized English-language studies directly relevant to acute trauma care and addressing clinically meaningful applications across the trauma pathway, including prehospital triage, diagnostic support, transfer decision-making, outcome prediction, and follow-up care. As this article is a mini-review rather than a systematic review, the goal was not exhaustive retrieval but a focused synthesis of representative and clinically relevant recent evidence.

## 2 The evolving landscape of intelligent technologies in emergency trauma care: from prehospital to long-term outcomes

### 2.1 Artificial intelligence: from point-of-injury triage to automated diagnostics and outcome prediction

The application of AI in trauma care spans the entire patient journey, beginning at the point of injury (1). Its core value lies in processing multifaceted data streams, vital signs, injury patterns, imaging pixels, and laboratory results, to extract predictive signals that may elude conventional clinical assessment or scoring systems (6, 7).

#### 2.1.1 Prehospital triage and early risk stratification

Effective field triage is paramount for directing patients to appropriate care facilities, yet traditional tools like the American College of Surgeons field triage guidelines have recognized limitations in sensitivity (11). Machine learning models are being developed to enhance this process by incorporating a wider array of early data points. Studies demonstrate that ML algorithms can analyze prehospital physiological features, such as waveforms and derived vital sign patterns, to predict the need for lifesaving interventions (LSIs) with significant accuracy (12). For instance, an ensemble ML model using data from the first 15 min of care achieved an area under the receiver operating characteristic curve (AUC) of 0.810 for predicting LSI delivery, offering a potential decision-support tool for medics in austere environments (12).

Further development focuses on creating interpretable and practical tools for prehospital use. The Grade for Interpretable Field Triage (GIFT) score, developed using an interpretable ML framework on Asian trauma registry data, effectively predicted emergency department mortality, highlighting age and vital signs as key determinants (13). Similarly, other research has constructed models to predict severe trauma (Injury Severity Score ≥16) and critical resource use based on prehospital variables, aiming to achieve an undertriage rate of less than 10% while enhancing the performance of existing guidelines (11). For specific injury mechanisms, specialized models show promise. A deep neural network trained to predict shock, need for massive transfusion (MT), and major surgery in patients with truncal gunshot wounds reported high predictive accuracy (AUCs 0.82–0.89) and incorporated a confidence estimation feature to guide triage certainty (14).

Across prehospital studies, a consistent pattern is that machine-learning models can enhance early risk stratification beyond rule-based triage by incorporating multidimensional physiologic and contextual inputs; however, most evidence still derives from retrospective datasets, and prospective validation within operational trauma systems remains limited.

#### 2.1.2 Diagnostic imaging support and workflow optimization

The emergency department, particularly in trauma centers, generates a high volume of time-critical imaging studies. AI, especially convolutional neural networks (CNNs), is demonstrating strong capabilities in assisting with rapid image interpretation, thereby addressing radiologist workload and potentially reducing diagnostic delays (15, 16). A prominent application is the automated detection of intracranial hemorrhage (ICH) on non-contrast head CT scans. Commercially available algorithms can prioritize studies with a high probability of ICH in the reading queue. The implementation of such a system has been associated with a statistically significant reduction in report turnaround time and, in one study, with significant reductions in 30- and 120-day all-cause mortality and morbidity for ICH patients, suggesting that expedited diagnosis translates to better outcomes (16, 17). Importantly, real-world external validation studies confirm that well-trained models maintain high performance (AUC > 0.95) on temporally and geographically distinct datasets, a crucial step toward demonstrating generalizability (18).

The utility of CNNs extends to fracture detection across multiple anatomical sites. Algorithms have shown high sensitivity and specificity in detecting fractures of the wrist, ribs, pelvis, and spine on both radiographs and CT scans (19–22). For rib fractures, which are frequently missed on initial chest radiographs, AI assistance has been shown to improve the sensitivity of detection, particularly for less experienced clinicians (19, 21, 23). In pediatric trauma, evaluation of commercial AI algorithms for peripheral limb fracture detection on radiographs revealed acceptable and consistent performance, with the potential to improve diagnostic accuracy for less-experienced residents (24). Beyond detection, AI models are advancing to more complex tasks such as injury grading. An award-winning model from the Radiological Society of North America (RSNA) Abdominal Trauma AI Challenge demonstrated strong external validation performance (AUC 0.931) for detecting and grading splenic injuries on CT, indicating progress toward nuanced diagnostic support (25). Furthermore, multimodal models that integrate imaging data with unstructured clinical text are emerging. The SMART model, which combines non-contrast CT analysis with narrative text processing using large language model embeddings, achieved high sensitivity for abdominal solid organ injury screening, highlighting a path toward more comprehensive AI diagnostic assistants (26).

Taken together, the imaging literature suggests that AI is currently most mature as a workflow-augmentation tool for prioritization and early detection, but gains in diagnostic speed should not be assumed to translate automatically into improved patient outcomes without prospective implementation evidence.

#### 2.1.3 Prediction of clinical trajectories and specific outcomes

A rapidly expanding area of AI application is prognostic modeling, moving beyond diagnosis to forecast a patient’s clinical course (5, 6). These models aim to support clinical decision-making for resource allocation and personalized intervention strategies. Mortality prediction is a common focus, with numerous studies developing ML models that outperform traditional scoring systems like the Injury Severity Score (ISS) or triage scales. For example, an AI model using nationwide Korean emergency department data achieved an exceptional AUC of 0.997 for predicting trauma mortality, identifying age, unresponsiveness, and specific injury patterns as top predictors (27). Ensemble ML approaches that integrate early vital signs and laboratory data have also shown excellent discrimination (AUC up to 0.951) for in-hospital mortality in severely injured patients (28). To enhance clinical usability, interpretable AI models have been packaged into point-of-care tools. The Trauma Outcome Predictor (TOP) is a smartphone application based on Optimal Classification Trees that provides nonlinear risk estimation for mortality and complications with high accuracy, facilitating bedside counseling (29). Similarly, the TROUT Index uses an explainable ML framework to create a frailty assessment tool for older trauma patients, prognosticating adverse outcomes like mortality and prolonged hospitalization (30).

Prediction modeling is also being applied to specific critical interventions. Forecasting the need for massive transfusion is vital for activating hemorrhage protocols promptly (31, 32). Multiple ML models have been developed for this purpose, with several showing good to excellent discrimination (AUC > 0.8) using early clinical data available in the prehospital setting or emergency department (31, 33). Advanced models now aim to predict not just the binary need for transfusion but also the optimal amount and combination of blood products, leveraging multinational registry data for robust training (34). Beyond immediate physiological outcomes, AI is increasingly used to predict psychological sequelae. Connectome-based predictive modeling using functional MRI data acquired 1 month post-trauma has been shown to predict the severity of post-traumatic stress disorder (PTSD) symptoms up to 14 months later, identifying specific neural networks associated with symptom trajectories (35). Simpler, scalable tools are also being developed for acute care settings, such as a brief 5-item ML-derived screener from the Immediate Stress Reaction Checklist, which demonstrated good accuracy (AUC 0.84) for identifying patients at risk of chronic PTSD (36).

Overall, prognostic AI models are increasingly capable of identifying patients at risk for death, hemorrhage, prolonged hospitalization, and psychological sequelae. Yet, cross-study comparison remains difficult because of heterogeneous endpoints, inconsistent calibration reporting, and limited evidence that these tools meaningfully change bedside decisions or downstream outcomes.

### 2.2 Telemedicine: bridging geographical gaps and optimizing resource utilization

While AI excels at data analysis and pattern prediction, telemedicine addresses the challenge of geographical and expertise distribution (4). It enables the virtual presence of specialist knowledge, thereby supporting trauma care in resource-limited or remote settings (8).

#### 2.2.1 Virtual triage and transfer decision support

A significant driver for telemedicine in trauma is the reduction of medically unnecessary interfacility transfers (10). Studies from Level I trauma centers consistently report that a substantial proportion of transferred patients, often between 13 and 27%, are discharged from the emergency department without requiring admission or complex intervention, indicating potential overtriage (10, 37). These patients frequently present with isolated injuries to anatomical regions like the hand, face, or ophthalmology, where referring facilities may lack specific diagnostic or management capabilities (10, 38). Telemedicine offers a solution by allowing remote specialists to visually assess injuries, review imaging, and guide local management. The implementation of dedicated programs, such as a hand trauma telemedicine system, has led to a statistically significant decrease in unnecessary transfers and associated transportation costs, as a higher percentage of transferred patients subsequently required admission for necessary care (38). In pediatric trauma, a “Virtual Pediatric Trauma Center” model is being prospectively evaluated to determine if telemedicine consultation can maintain patient safety while improving family-centered outcomes and reducing transfer rates compared to standard telephone consultation (39).

Collectively, these studies suggest that telemedicine has its clearest current value in refining transfer decisions and reducing avoidable interfacility movement, although the magnitude of benefit remains dependent on local case mix, specialist availability, and network infrastructure.

#### 2.2.2 Remote specialist consultation and management guidance

Telemedicine facilitates not only triage decisions but also active guidance for diagnosis and management at the referring site (9). This can range from assisting with the interpretation of imaging studies to providing real-time procedural advice. In trauma care, this often involves remote surgical or critical care consultation. Evidence suggests that telemedicine is preferentially used for more severely injured patients and may support procedural interventions at remote sites, such as guiding complex laceration repairs or joint reductions, which might otherwise necessitate transfer (9, 10). For orthopedic injuries, virtual fracture clinics have gained traction, especially accelerated by the COVID-19 pandemic. These protocols allow for the remote follow-up of patients with simple fractures using video calls, often led by physiotherapists or advanced practice providers. Studies indicate that such virtual clinics are safe, do not increase complication rates, and are highly acceptable to patients (40, 41). Furthermore, they can significantly improve healthcare efficiency by reducing the number of in-person follow-up appointments and radiographic imaging, while optimizing surgery scheduling and decreasing emergency department reattendances (42).

The clinical effectiveness of telemedicine in trauma appears context-dependent. A systematic review and meta-analysis found that telemedicine was associated with higher recorded Injury Severity Scores and increased blood transfusion rates, likely reflecting improved detection of severe injuries and more accurate triage facilitated by specialist input. However, no statistically significant impact on overall mortality, transfer times, or emergency department utilization was conclusively demonstrated, pointing to the influence of heterogeneous protocols and case mix (8). Satisfaction among both patients and physicians with telemedicine services is generally reported to be high (8). In specialized contexts like follow-up care for survivors of sexual assault and intimate partner violence, qualitative studies indicate telemedicine is feasible and acceptable, offering increased comfort and convenience, though safety and privacy concerns must be carefully addressed on an individual basis (43).

## 3 Gaps, controversies, and implementation challenges

Despite the promising advancements, the integration of smart healthcare into mainstream trauma care faces significant hurdles (3, 5). These span the spectrum from the quality of the underlying evidence to profound ethical questions and practical integration barriers.

### 3.1 Evidence gaps: from retrospective validation to prospective clinical utility

A predominant limitation in the current literature is the heavy reliance on retrospective study designs. Scoping reviews consistently note that the vast majority of AI applications in emergency and trauma care are validated using historical data, with a striking paucity of prospective controlled trials or real-world interventional studies (3, 15, 44). While retrospective analyses are essential for proof-of-concept and model development, they are insufficient to prove clinical utility, the measurable improvement in patient outcomes or system efficiency when the tool is deployed in practice (6). For instance, many high-performing prediction models for mortality or transfusion exist *in silico*, but very few have undergone rigorous external validation in prospective cohorts, and even fewer have been tested for their impact on clinician behavior or patient trajectories (32, 33). Similarly, for telemedicine, while numerous descriptive studies exist, there is a recognized scarcity of articles providing robust quantitative assessment of its impact on hard clinical endpoints like diagnostic accuracy or mortality (9). The most significant literature gap identified for generative AI in trauma is the near-absence of treatment-oriented applications that provide real-time guidance for urgent interventions, as opposed to analytical or diagnostic support (15). This evidence chasm between technical validation and proven clinical benefit represents the single greatest barrier to widespread adoption.

### 3.2 Methodological and ethical controversies: the “black box” dilemma and algorithmic bias

The opacity of many complex AI models, particularly deep learning algorithms, creates a “black box” problem that erodes clinician trust and poses ethical challenges (5, 45). If a model recommends against a transfer or predicts a low risk of hemorrhage, clinicians need to understand the rationale to reconcile it with their own assessment. The field is responding with a push toward explainable AI (XAI). Studies are increasingly incorporating techniques like Shapley Additive exPlanations (SHAP) analysis to illuminate which features (e.g., D-dimer levels, Glasgow Coma Scale score) most influence a model’s prediction (46, 47). The development of inherently interpretable models, such as Optimal Classification Trees used in the Trauma Outcome Predictor, represents another approach to maintaining transparency (29). The goal is to shift from pure automation to meaningful augmentation, where AI supports cognitive work under pressure without usurping clinical judgment (45).

Closely linked to transparency is the risk of algorithmic bias. AI models learn patterns from training data, and if that data reflects historical healthcare disparities (e.g., underdiagnosis in certain demographic groups, unequal access to care), the model may perpetuate or even amplify these biases. This raises profound ethical concerns regarding equitable care. Furthermore, the calibration of models is crucial; a model that accurately ranks patient risk (good discrimination) may systematically over- or underestimate the absolute probability of an event (poor calibration), leading to misguided clinical decisions. This calibration gap is noted as a particular issue in some injury scoring models, which can permit dangerous false negatives (15). Ensuring that AI tools are fair, accountable, and validated across diverse populations is a non-negotiable prerequisite for clinical use.

### 3.3 Integration barriers: workflow, infrastructure, and human factors

Successful implementation extends beyond algorithmic performance. Seamless integration into high-stakes, fast-paced clinical workflows is a formidable challenge (5). AI alerts or telemedicine consoles must provide information without adding to cognitive load or interrupting critical tasks. Human factors engineering is essential; poorly designed interfaces can lead to alert fatigue, automation bias (over-reliance on the tool), or outright rejection by clinical staff (5, 45). Infrastructure demands are also substantial. AI imaging analysis requires significant computational resources and seamless integration with picture archiving and communication systems (PACS). Telemedicine depends on reliable, high-bandwidth connectivity and appropriate audiovisual equipment at both hub and spoke sites. An observational study of an AI rib fracture detection system noted a 14-day outage due to graphics processing unit failure, underscoring the importance of resilient technical infrastructure (23). Finally, regulatory and reimbursement landscapes for both AI and telemedicine are still evolving, creating uncertainty for healthcare institutions. Overcoming these barriers requires close collaboration between data scientists, clinicians, informaticians, and healthcare administrators to design systems that are not only intelligent but also usable, reliable, and sustainable (6).

## 4 Conclusion and future directions

The integration of smart healthcare technologies into emergency trauma care is undeniably advancing from conceptual promise toward tangible, albeit incremental, clinical reality (3, 7). Artificial intelligence has demonstrated robust capabilities in enhancing prehospital triage accuracy, accelerating and refining diagnostic imaging interpretation, and providing sophisticated prognostic forecasts for mortality, resource need, and psychological outcomes (1, 6). In parallel, telemedicine has established its value as a strategic tool for bridging geographical care gaps, supporting appropriate triage and transfer decisions, and enabling remote specialist guidance, thereby optimizing the use of finite trauma system resources (4, 8). Together, these technologies are poised to transform the trauma care continuum into a more data-informed, efficient, and patient-centric system. From a clinical perspective, the relevance of these technologies lies not only in predictive accuracy but in whether they improve concrete decisions along the trauma pathway, including transport destination, imaging prioritization, hemorrhage-pathway activation, transfer avoidance, and post-discharge follow-up planning.

However, the path to realizing this potential is obstructed by significant challenges that must be deliberately addressed. The foremost priority is to transition from a plethora of retrospective validations to a robust evidence base derived from prospective, controlled interventional trials (6, 44). The ultimate measure of success for any AI tool or telemedicine protocol is not its area under the curve on a historical dataset, but its demonstrable impact on patient outcomes, clinician decision-making, and system efficiency when deployed in real-world settings (1, 45). Future research must rigorously evaluate these technologies not as isolated algorithms but as integrated components of clinical workflows, assessing their effect on tangible endpoints such as time to definitive care, complication rates, functional recovery, and cost-effectiveness. Accordingly, future studies should evaluate these tools as components of clinical workflows rather than as isolated algorithms, with attention to decision quality, timeliness of definitive care, functional recovery, and cost-effectiveness.

Simultaneously, the field must confront and mitigate the ethical and methodological controversies inherent to advanced AI. The development and adoption of explainable AI frameworks are non-negotiable for building clinician trust and ensuring safe, accountable decision support (29, 45). Proactive efforts to audit and debias algorithms across diverse populations are essential to prevent the perpetuation of healthcare disparities. Furthermore, the focus should increasingly shift from pure automation to human-centered augmentation. The most valuable role for AI in trauma is likely as a cognitive aid that processes complex data streams in the background, highlights critical patterns, and presents actionable insights, thereby preserving clinicians’ cognitive capacity for integrative judgment and patient communication (7, 45).
